# Abnormal lipid profiles are associated with markers of impaired fibrinolysis in patients with cirrhosis

**DOI:** 10.1016/j.rpth.2026.106975

**Published:** 2026-09-18

**Authors:** Fynn L. Elvers, Jelle Adelmeijer, Marilena Stamouli, William Bernal, Vishal C. Patel, Ton Lisman

**Affiliations:** 1 Surgical Research Laboratory and Section of Hepatobiliary Surgery and Liver Transplantation, Department of Surgery, University Medical Center Groningen, University of Groningen, Groningen, the Netherlands; 2 Roger Williams Institute of Liver Studies, School of Immunology & Microbial Sciences, Faculty of Life Sciences and Medicine, King’s College London, Foundation for Liver Research and King’s College Hospital, London, UK; 3 Liver Intensive Therapy Unit, Institute of Liver Studies, King’s College Hospital, Denmark Hill, London, UK

**Keywords:** apolipoprotein A-I, dyslipidemias, fibrin clot lysis time, fibrinolysis, liver cirrhosis, sepsis, thrombosis, triglycerides

## Abstract

**Background:**

Patients with liver cirrhosis exhibit abnormalities in lipid metabolism and in circulating proteins of clot breakdown. Recent studies have shown relationships between dyslipidemia and fibrinolytic abnormalities in noncirrhotic populations.

**Objectives:**

We investigated associations between lipid profiles and fibrinolytic markers in patients with cirrhosis.

**Methods:**

This observational study analyzed samples from 147 patients with stable cirrhosis, decompensated cirrhosis, acute-on-chronic liver failure, and sepsis without pre-existing chronic liver disease, and from 38 healthy controls. We studied fibrinolysis by clot lysis time (CLT), plasminogen, plasminogen activator inhibitor-1 (PAI-1), and tissue-type plasminogen activator (tPA), and lipid profiles by apolipoproteins A1 and B, common lipoproteins, triglycerides, and total cholesterol.

**Results:**

CLT was slightly enhanced in stable and decompensated cirrhosis, but prolonged in acute-on-chronic liver failure and sepsis. Plasminogen levels decreased, whereas PAI-1 and tPA increased with cirrhosis severity. All lipid parameters decreased with cirrhosis severity, besides triglyceride levels, which were preserved. In an analysis of all patients with cirrhosis combined, apolipoprotein A1 correlated inversely with CLT, levels of PAI-1 and tPA, and positively with plasminogen. Apolipoprotein B, low-density lipoprotein, and lipoprotein(a) correlated positively with plasminogen. Triglycerides correlated positively with CLT.

**Conclusion:**

Cirrhosis is characterized by decreased (apo)lipoprotein levels, preserved triglyceride levels, and a shift from enhanced to impaired fibrinolysis with increasing disease severity. Lower apolipoprotein A1 levels and higher triglyceride levels are associated with impaired fibrinolysis, which may reflect biological interactions between lipid metabolism and fibrinolysis or parallel alterations secondary to hepatic dysfunction.

## Essentials

• Liver cirrhosis can lead to the development of dangerous blood clots, but treatment options are limited.
• We measured levels of blood lipids and markers of clot breakdown in patients with cirrhosis.
• Low apolipoprotein A1 and high triglyceride lipid levels are linked to impaired blood clot breakdown.
• These links may reflect biological interactions or unrelated parallel consequences of cirrhosis.

## Graphical abstract

**Figure:**
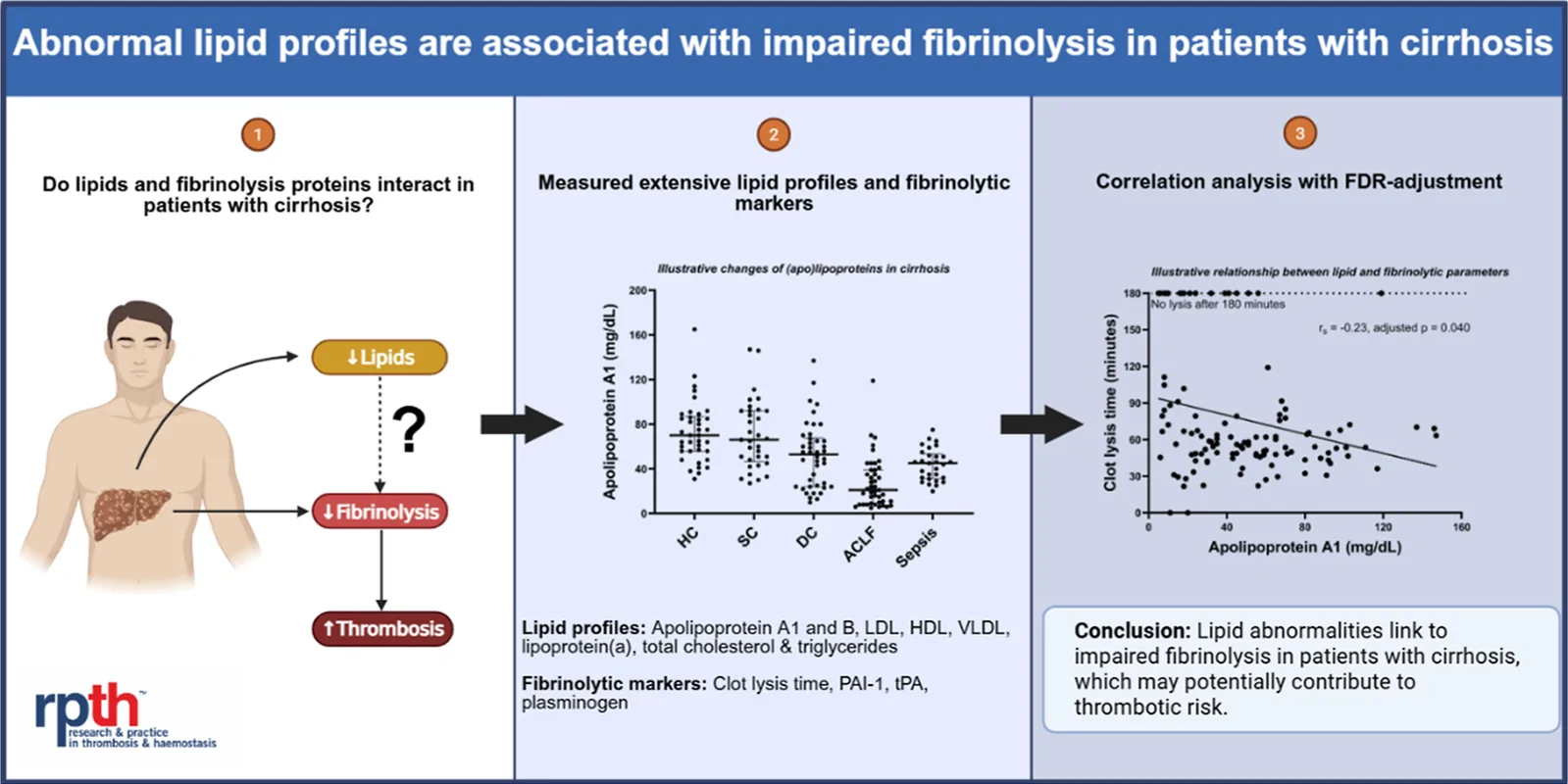

## 1 Introduction

Patients with cirrhosis show profound abnormalities in their hemostatic system [1]. Fibrinolysis is the final stage of hemostasis, in which tissue-type plasminogen activator (tPA) converts plasminogen into plasmin to degrade fibrin clots, whereas plasminogen activator inhibitor-1 (PAI-1) inhibits tPA activity. Cirrhosis is associated with altered plasma levels of fibrinolytic proteins. Endothelial activation secondary to portal hypertension increases tPA and PAI-1 release [2,3], whereas impaired hepatocellular synthetic function reduces circulating levels of plasminogen, α2-antiplasmin, and thrombin activatable fibrinolysis inhibitor [3]. Clot lysis time (CLT), a plasma-based assay reflecting fibrinolytic capacity, indicates slightly enhanced fibrinolysis in early cirrhosis but impaired fibrinolysis in advanced cirrhosis [4]. Prolonged CLT is associated dose-dependently with increased risk of venous thromboembolism in patients without cirrhosis [5]. Therefore, impaired fibrinolysis may contribute to thrombosis risk in patients with advanced cirrhosis.

In fact, cirrhosis is associated with an elevated risk of developing venous thromboembolism and portal vein thrombosis [6,7]. Thrombosis may accelerate liver disease progression and elevate morbidity and mortality before and after liver transplantation [[7], [8], [9]]. Despite these complications, current anticoagulant management of thrombosis remains suboptimal. Limitations include a lack of high-quality evidence, low prescription rates, underdosing, and discontinuation due to concerns about bleeding side effects [[10], [11], [12]]. Consequently, there is a need to identify additional pathways contributing to thrombotic risk that may be amenable to therapeutic intervention.

Abnormal lipid parameters represent a potential modulator of fibrinolysis and thrombosis. In noncirrhotic populations, dyslipidemia is associated with increased venous thromboembolism risk [13,14], and lipid-lowering therapies such as statins attenuate this thrombotic risk [15]. Emerging evidence suggests that abnormal lipid levels affect thrombosis through direct interactions with the fibrinolytic system [16]. For example, elevated triglycerides are associated with increased PAI-1 and tPA levels [17]. In contrast, lower levels of high-density lipoprotein (HDL) and apolipoprotein A1 are associated with reduced clot permeability and a prolonged CLT [18], and lipoprotein(a) prolongs *ex vivo* CLT [19]. Experimental studies support these associations, as triglyceride-rich very-low-density lipoprotein (VLDL) increases PAI-1 synthesis in HepG2 cells [20], apolipoprotein A1 deficiency in mice results in a prothrombotic state due to lack of endothelial nitric oxide synthase induction [21], and lipoprotein(a) impairs plasminogen binding to fibrin [22]. Together, these findings support the notion that lipid abnormalities may influence fibrinolytic capacity.

Lipid abnormalities frequently occur in cirrhosis, as hepatocellular injury disrupts lipoprotein synthesis, secretion, and clearance, resulting in decreased lipid and lipoprotein levels [23]. At the same time, metabolic dysfunction-associated steatotic liver disease (MASLD) and alcohol-related liver disease may be associated with increases in atherogenic lipids and lipoproteins [24,25]. Despite these prominent alterations, it is currently unclear whether abnormal lipid parameters are associated with fibrinolytic abnormalities in patients with cirrhosis. This gap is clinically relevant because current antithrombotic management of patients with cirrhosis is suboptimal [1]; lipid-modifying therapies are widely available and may improve fibrinolysis to reduce thrombotic risk [15].

Therefore, this study aimed to (1) quantify fibrinolytic capacity and (2) lipid profiles, and (3) assess their relationship in patients with cirrhosis compared with healthy controls. We hypothesized that cirrhosis is associated with disease severity–dependent impairment of fibrinolysis and altered lipid profiles, with apolipoprotein B–containing lipoproteins associated inversely and apolipoprotein A1–containing lipoproteins associated positively with fibrinolysis.

## 2 Methods

### 2.1 Study design, setting, and participants

This study was designed as a cross-sectional analysis of a subset of banked serum and citrated platelet-poor plasma samples nested within a single-center prospective longitudinal observational cohort study. We included patients with cirrhosis or sepsis without pre-existing chronic liver disease consecutively enrolled between May 2018 and March 2024 in the ongoing “Gut-liver axis in liver disease and liver transplantation” study from the outpatient clinic, hospital wards, or the liver intensive care unit of King’s College Hospital, a 950-bed tertiary center in London, United Kingdom. Ethical approval was granted by the National Research Ethics Service Committee London—Westminster (study number 12/LO/1417) in accordance with the Declaration of Helsinki and Istanbul. Written informed consent was provided by participants or personal consultees in case of mental incapacity. This study was conducted and reported in accordance with the STROBE (Strengthening the Reporting of Observational Studies in Epidemiology) guidelines.

Patients were eligible for inclusion if they had cirrhosis, defined by at least 2 of the following: histologic evidence of cirrhosis on liver biopsy, laboratory findings indicative of cirrhosis, or radiological signs of cirrhosis or portal hypertension. Patients were stratified into clinical phenotypes of stable cirrhosis, decompensated cirrhosis, or acute-on-chronic liver failure (ACLF). Stable cirrhosis was defined as cirrhosis without signs of hepatic decompensation. Decompensated cirrhosis was defined as the onset of ascites, jaundice, hepatic encephalopathy, gastrointestinal bleeding, bacterial infection, or any combination thereof, in patients with pre-existing cirrhosis. ACLF was defined according to the CANONIC (Causal Assessment of Acute-on-Chronic Liver Failure) study as acute decompensation in the presence of organ failure determined by the Chronic Liver Failure Consortium organ failure criteria across the liver, kidney, brain, coagulation, circulatory, and respiratory systems. Given the extensively shared features of systemic inflammation and organ failure between patients with ACLF and sepsis [26], we included patients with sepsis based on the “Sepsis-3 criteria” as a positive disease control to discern the effects of critical illness and liver disease on hemostatic abnormalities in patients with ACLF. Exclusion criteria for all patients were age below 18 years or above 75 years, pregnancy, congenital coagulation disorders, extrahepatic malignancy, hepatocellular carcinoma, or replicating human immunodeficiency virus.

A formal a priori sample size calculation could not be performed because, at the time of study design, comprehensive data on comparable patient populations and effect sizes relevant to the specific research question were not available. Therefore, a pragmatic sample size target of at least 30 patients per group was selected based on the availability of sufficient plasma and serum sample volumes at the start of the analyses.

To establish reference values for the various assays performed, we included 38 healthy controls aged >18 years at King’s College Hospital between July 2017 and March 2024. Exclusion criteria were pregnancy, active breastfeeding, history of thrombotic, hematological or liver disease, and active use of anticoagulants, antiplatelets, or oral contraceptives.

Demographic, clinical, and laboratory data were recorded prospectively at the time of enrollment in the “Gut-liver axis in liver disease and liver transplantation” study. Demographic data included age, sex, and ethnicity. Clinical data were recorded for disease etiology, physical examination and vital parameters on admission, ascites grade, hepatic encephalopathy grade, and organ failures. In addition, the following clinical composite scores were calculated: Child-Pugh-Turcotte; Model of End Stage Liver Disease; Chronic Liver Failure Consortium-Organ Failure, Chronic Liver Failure Consortium-Acute decompensation, and Chronic Liver Failure Consortium-Acute-on-Chronic Liver Failure scores; and Sequential Organ Failure Assessment. Laboratory data were recorded on hematology and biochemistry.

### 2.2 Blood sampling

Patient blood samples were drawn by peripheral venous phlebotomy, from a nonheparinized central venous catheter, or from an arterial line within 2 days after admission into 3.2% sodium citrate tubes. Collection was completed before the administration of blood products, anticoagulants, or antiplatelet medications. Blood samples from healthy volunteers were drawn by peripheral venous phlebotomy. Samples were processed to platelet-poor plasma within 2 hours of collection through double centrifugation at 12 °C at 2000 *g* and 10,000 *g* for 10 minutes each, with subsequent aliquoting and storage at –80 °C. To attain serum, peripheral blood was collected in serum clot activator tubes, centrifuged at 5000 *g* for 10 minutes at 4 °C, and immediately stored at –80 °C until further use.

### 2.3 CLT assay

To estimate global fibrinolytic capacity, we conducted a plasma-based CLT assay as described previously [5]. Lysis of a tissue factor–induced clot by exogenous tPA was studied by observing changes in turbidity. We prepared a mix of tPA (Actilyse; Boehringer Ingelheim; final concentration 56 ng/mL), tissue factor (Dade Innovin; Siemens Healthcare; final dilution 1:1000), calcium chloride (final concentration 17 mM), phospholipids (consisting of 40% l-α-dioleoylphosphatidylcholine, 20% l-α-dioleoylphosphatidylserine, and 40% l-α-dioleoylphosphatidylethanolamine, all from Avanti Polar Lipids; final concentration 10 μM), and Hepes buffer (25 mM Hepes, 137 mM NaCl, 3.5 mM KCl, 0.1% bovine serum albumin, pH 7.4). The mix was added to patient plasma (1:1 dilution), and changes in turbidity at 340 nm were measured (3 hours, 20-second intervals, 3 seconds of automatic shaking before assay start, 37 °C) on a SpectraMax 340 kinetic microplate reader using SoftMax Pro software (Molecular Devices Corporation). CLT was defined as the time between 50% coagulation and 50% fibrinolysis, and analyzed using LysisAnalyzer software (version 4.0.0.0). Samples were arbitrarily assigned a CLT of 180 minutes if lysis was incomplete after 3 hours.

### 2.4 Fibrinolytic protein assays

Plasma plasminogen activity levels were determined on an automated coagulation analyzer (StaCompact3) using reagents and protocols of the manufacturer (Stago). Antigen levels of tPA and PAI-1 were measured using commercially available enzyme-linked immunosorbent assays (Zymutest tPA Antigen, Hyphen BioMed; and DuoSet, R&D Systems).

### 2.5 Lipid profile assays

Serum lipid parameters were quantified using commercially available assays with the respective manufacturers' instructions and reagents. We measured concentrations of apolipoprotein B (Immunoturbidimetric assay, Atellica CI Analyzer; Siemens), apolipoprotein A1 (Duoset ELISA kit, DY3664-05; Bio-Techne), HDL cholesterol (Colorimetric Assay Kit, EEA012; Thermo Fisher), LDL cholesterol (Colorimetric Assay Kit, EEA014; Thermo Fisher), VLDL (ELISA Kit, abx153487; Abbexa), lipoprotein(a) (Colorimetric ELISA Kit, NBP2-60510; Novus Biologicals), total cholesterol (Colorimetric Assay Kit, NBP3-25834; Novus Biologicals), and triglycerides (Colorimetric Assay Kit, NBP3-24540; Novus Biologicals).

### 2.6 Statistical analysis

Analysis was performed using Prism (version 10.5.0; GraphPad Software for Microsoft Windows) and IBM Statistical Package for the Social Sciences (version 30.0; IBM). Gaussian distribution of continuous variables was assessed using the Shapiro-Wilk test and visual inspection of histograms and Q-Q plots. Continuous variables and ordinal variables, treated as scores, were reported as mean ± SD or median [IQR], as appropriate. Categorical variables were expressed as counts with corresponding percentages (%). For within-group comparisons across multiple conditions, the Kruskal-Wallis test with Dunn’s multiple comparisons test and Brown-Forsythe and Welch’s 1-way analysis of variance with Dunnett’s T3 multiple comparisons test were used for nonparametric and parametric data, respectively. Associations between continuous variables were assessed using Spearman’s rank correlation coefficient ρ (*r*_s_). False discovery rate correction using the Benjamini, Krieger, and Yekutieli method (*Q* = 0.05) was applied for the predefined correlation analysis to account for multiple comparisons and reported as adjusted *P* values corresponding to Spearman’s ρ (*r*_s_). Statistical significance was defined as a 2-sided *P* value of <.05. Variables with missing data were not part of any statistical analyses.

## 3 Results

### 3.1 Patient characteristics

We analyzed plasma and serum samples from 185 participants, including 33 patients with stable cirrhosis, 42 with decompensated cirrhosis, 43 with ACLF, 29 with sepsis without pre-existing chronic liver disease, and 38 healthy controls. Demographic, clinical, and laboratory characteristics are summarized in Tables 1 and 2. Briefly, among patients and controls, 47 (32%) and 23 (62%) were female, respectively. Mean age was 55.2 ± 11.2 years in patients, compared with 36.0 ± 10.6 years in controls. Median body mass index was 27.0 [22.9-31.1] in patients and 24.9 [21.6-29.1] in controls. Among patients with cirrhosis, 35 patients (30%) were Child-Turcotte-Pugh class A, 35 (30%) class B, and 47 (40%) class C, and the median Model of End-Stage Liver Disease score was 16.0 [10.0-27.0]. Cirrhosis etiologies included alcohol-related liver disease in 72 patients (60%), MASLD in 22 (18%), hepatitis C virus infection in 7 (6%), primary sclerosing cholangitis in 7 (6%), and other rare causes in 12 (10%). Data were missing in <5% for age, alanine aminotransferase, body mass index, Chronic Liver Failure Consortium-Acute-on-Chronic Liver Failure, C-reactive protein, creatinine, ethnicity, γ-glutamyl transferase, hepatic encephalopathy, sex, and urea. In addition, use of statins was unknown in 67 (45.5%) patients; however, statin use occurred only in 10 (12.5%) of the remaining 80 (54.5%) patients.

**Table 1:** Demographic and clinical data of patients and controls.

Variable	Healthy controls (*n* = 38)	Stable cirrhosis (*n* = 33)	Decompensated cirrhosis (*n* = 42)	Acute-on-chronic liver failure (*n* = 43)	Sepsis without pre-existing liver disease (*n* = 29)
Demographics^a^					
Age (y)	36.0 ± 10.6	60.8 ± 7.8	57.4 ± 8.7	49.0 ± 12.3	54.9 ± 12.1
Male	14 (38)	28 (85)	27 (64)	27 (63)	18 (62)
Female	23 (62)	5 (15)	15 (36)	16 (37)	11 (38)
Ethnicity					
White	26 (70)	28 (85)	36 (85)	34 (79)	17 (59)
Asian	8 (21)	2 (6)	2 (5)	5 (12)	0 (0)
Black	2 (5)	3 (9)	2 (5)	0 (0)	11 (38)
Other ethnicity	1 (3)	0 (0)	2 (5)	4 (9)	1 (3)
Cirrhosis etiology					
Alcohol-related	n/a	19 (58)	21 (50)	32 (74)	n/a
Autoimmune hepatitis	n/a	0 (0)	1 (2)	1 (2)	n/a
Biliary atresia	n/a	0 (0)	0 (0)	1 (2)	n/a
Budd-Chiari syndrome	n/a	0 (0)	1 (2)	0 (0)	n/a
Cryptogenic	n/a	0 (0)	2 (5)	0 (0)	n/a
Hemochromatosis	n/a	1 (3)	0 (0)	0 (0)	n/a
Hepatitis C virus	n/a	4 (12)	2 (5)	1 (2)	n/a
MASH/MASLD	n/a	7 (21)	11 (26)	4 (10)	n/a
Primary biliary cholangitis	n/a	0 (0)	2 (5)	0 (0)	n/a
Primary sclerosing cholangitis	n/a	1 (3)	2 (5)	4 (9)	n/a
Examination					
Body mass index (kg/m^2^)	24.9 [21.6-29.1]	28.4 [24.2-32.0]	28.1 [22.7-32.1]	24.5 [22.4-29.2]	26.2 [23.3-32.2]
Mean arterial pressure (mm Hg)	n/d	88.6 ± 12.6	84.5 ± 9.4	78.0 ± 11.8	78.5 ± 9.5
Ascites grade					
No	n/a	31 (94)	8 (20)	8 (19)	n/a
Slight	n/a	2 (6)	11 (27)	15 (35)	n/a
Moderate	n/a	0 (0)	22 (54)	20 (47)	n/a
Hepatic encephalopathy grade					
Grade 0	n/a	31 (94)	32 (76)	14 (33)	n/a
Grade 1	n/a	1 (3)	7 (16)	13 (31)	n/a
Grade 2	n/a	1 (3)	1 (2)	4 (10)	n/a
Grade 3	n/a	0 (0)	1 (2)	5 (12)	n/a
Grade 4	n/a	0 (0)	1 (2)	6 (14)	n/a
Child-Turcotte-Pugh class					
A	n/a	33 (100)	0 (0)	2 (5)	n/a
B	n/a	0 (0)	27 (64)	8 (22)	n/a
C	n/a	0 (0)	15 (35)	32 (68)	n/a
Scores					
Child-Turcotte-Pugh	n/a	5.4 ± 0.5	8.7 ± 1.4	10.5 ± 1.9	n/a
MELD	n/a	9.0 [7.0-11.0]	15.0 [11.0-19.0]	32.0 [22.0-36.0]	n/a
CLIF-C-AD	n/a	n/a	47.0 [42.0-51.0]	61.5 [52.0-65.0]	n/a
CLIF-C-ACLF	n/a	n/a	n/a	53.4 ± 11.6	n/a
CLIF-C-OF	n/a	6.0 [6.0-6.0]	6.0 [6.0-7.0]	10.0 [9.0-13.0]	n/a
SOFA	n/a	2.0 [2.0-5.0]	4.5 [2.0-6.0]	9.0 [7.0-13.0]	7.5 [4.5-12.0]
Interventions					
Vasopressors	n/a	0 (0)	0 (0)	17 (40)	19 (70)
Mechanical ventilation	n/a	0 (0)	1 (2)	18 (42)	18 (62)
Renal replacement therapy	n/a	0 (0)	0 (0)	21 (49)	12 (41)
Statin use^b^	n/a	5 (22)	2 (10)	0 (0)	3 (23)

Continuous variables are reported as mean ± SD or median [IQR] as appropriate, and categorical variables are presented as count (percentage).

CLIF-C, Chronic Liver Failure Consortium; CLIF-C-ACLF, Chronic Liver Failure Consortium-Acute-on-Chronic Liver Failure; CLIF-C-AD, Chronic Liver Failure Consortium-Acute Decompensation; CLIF-C-OF, Chronic Liver Failure Consortium-Organ Failure score; MASH/MASLD, metabolic dysfunction-associated steatohepatitis/metabolic dysfunction-associated liver disease; MELD, Model for End-Stage Liver Disease; n/a, not applicable; n/d, not determined; SOFA, Sequential Organ Failure Assessment.

a Demographic data were missing for *n* = 1 healthy control. Other ethnicities included Chinese or other than Chinese, White, Black, Asian or Mixed, or not stated.

b Statins included simvastatin, rosuvastatin, atorvastatin, and pravastatin. Other lipid-modulating drugs were not recorded. History on drug therapies was collected up to 3 days before and up to 7 days after the sampling date.

**Table 2:** Laboratory data of patients on admission.

Variable	Stable cirrhosis (*n* = 33)	Decompensated cirrhosis (*n* = 42)	Acute-on-chronic liver failure (*n* = 43)	Sepsis without pre-existing liver disease (*n* = 29)
Alanine aminotransferase (IU/L)	32.0 [20.0-45.0]	36.0 [22.5-56.0]	46.0 [28.5-81.0]	45.0 [19.0-143.0]
Albumin (g/L)	39.6 ± 5.6	31.9 ± 5.7	30.6 ± 6.0	26.4 ± 5.1
Alkaline phosphatase (IU/L)	99.0 [70.5-133.5]	131.5 [97.7-235.0]	122.0 [85.0-193.0]	86.0 [63.0-114.0]
Aspartate aminotransferase (IU/L)	36.0 [31.5-48.5]	58.5 [33.5-81.2]	91.0 [62.0-170.0]	71.0 [27.5-203.0]
Bilirubin, total (μmol/L)	18.0 [10.0-25.0]	36.5 [21.0-60.0]	206.0 [57.0-420.0]	11.0 [6.0-27.0]
Creatinine (μmol/L)	72.0 [62.5-93.5]	68.0 [56.7-87.2]	94.0 [67.5-159.5]	166.0 [69.5-256.5]
C-reactive protein (mg/L)	9.1 ± 13.3	17.1 ± 17.6	44.4 ± 38.1	218.2 ± 85.8
γ-Glutamyl transferase (IU/L)	105.0 [54.0-309.0]	80.0 [50.0-226.7]	89.0 [37.0-157.5]	57.0 [33.5-111.5]
Hemoglobin (g/dL)	122.7 ± 22.6	110.6 ± 20.3	88.7 ± 13.1	93.3 ± 16.9
International normalized ratio	1.1 [1.0-1.2]	1.3 [1.2-1.5]	1.7 [1.4-2.2]	1.2 [1.1-1.4]
Platelet count (×10^9^/L)	114.0 [89.5-173.0]	106.5 [69.0-179.5]	82.0 [55.0-116.0]	184.0 [108.5-244.5]
Urea (mmol/L)	5.3 [4.0-6.5]	5.3 [3.9-9.0]	8.7 [4.3-13.6]	10.1 [6.7-16.0]
White blood cell count (×10^9^/L)	5.2 [3.9-6.6]	4.7 [3.4-5.4]	10.1 [6.2-17.7]	16.1 [12.0-20.5]

Continuous variables are reported as mean ± SD or median [IQR] as appropriate, and categorical variables are presented as count (percentage). Laboratory data were not determined for healthy controls during inclusion.

### 3.2 Fibrinolytic capacity is normal to enhanced in stable and decompensated cirrhosis but impaired in ACLF and sepsis

To quantify fibrinolytic capacity and levels of fibrinolytic proteins, we measured plasma-based CLT and plasma levels of plasminogen, PAI-1, and tPA in patients and healthy controls (Figure 1). CLT was shorter in patients with stable cirrhosis and decompensated cirrhosis, indicating enhanced fibrinolysis. In contrast, CLT was prolonged in those with ACLF and sepsis, compared with controls, demonstrating impaired fibrinolysis. Notably, a few individual patients with stable cirrhosis and decompensated cirrhosis also displayed severely prolonged CLT. Plasminogen levels progressively decreased across cirrhosis severity, compared with controls, and were similar to levels in stable cirrhosis in patients with sepsis. PAI-1 levels were mildly higher in patients with stable cirrhosis and decompensated cirrhosis, and profoundly higher in patients with ACLF and sepsis than in controls. Similarly, tPA levels were mildly higher in patients with stable cirrhosis, decompensated cirrhosis, and sepsis, and profoundly higher in patients with ACLF than in controls.

**Figure 1:**
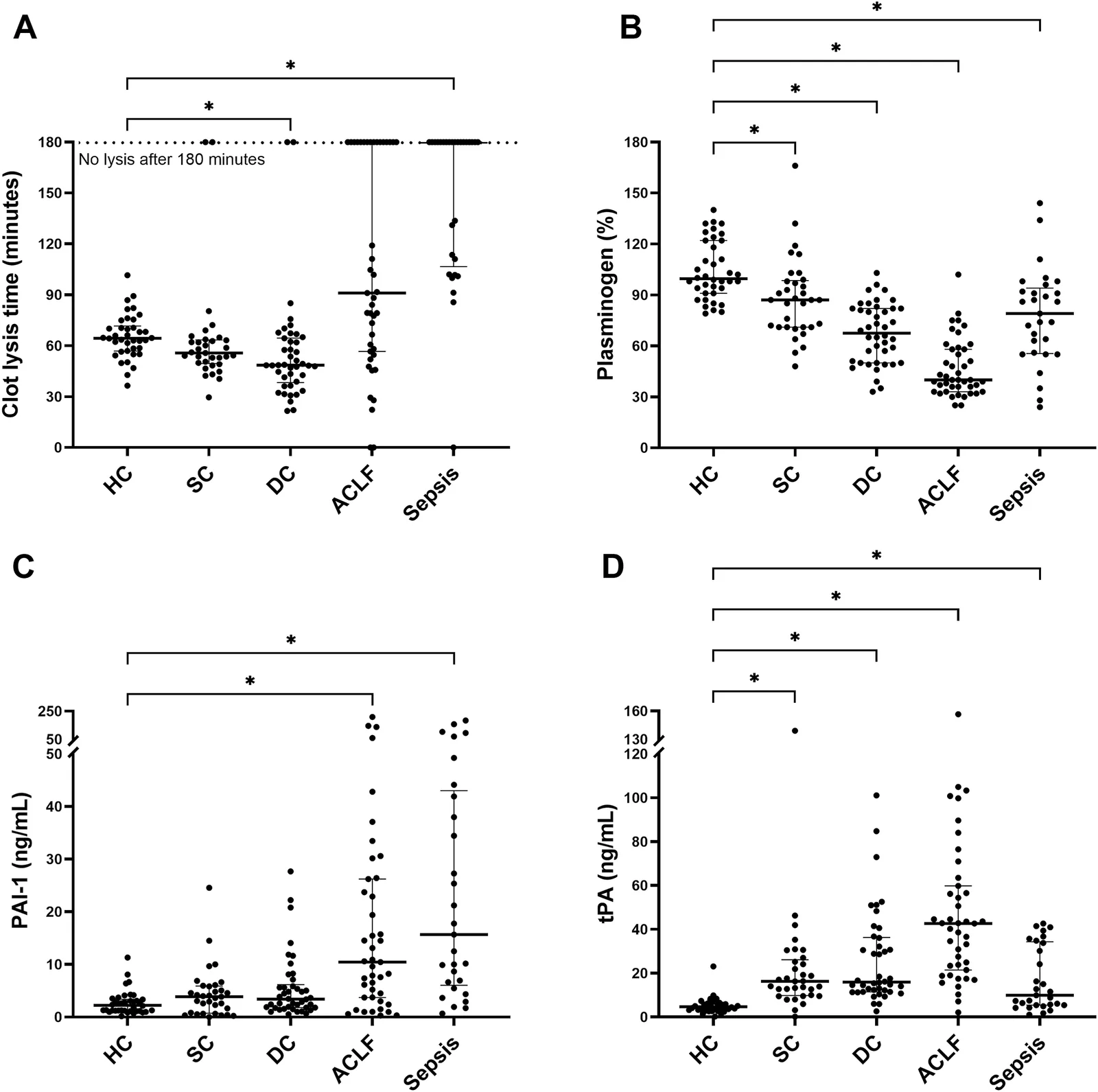
Plasma markers of fibrinolysis. Clot lysis time (A) and plasma levels of plasminogen (B), PAI-1 (C), and tPA (D) were measured in patients with stable cirrhosis (*n* = 33), decompensated cirrhosis (*n* = 42), ACLF (*n* = 43), and sepsis without pre-existing liver disease (*n* = 29), compared with healthy controls (*n* = 38). The bars and lines with error bars indicate medians with IQRs. ∗*P* < .05 all vs healthy controls using the Kruskal-Wallis test with Dunn’s multiple comparisons test. ACLF, acute-on-chronic liver failure; DC, decompensated cirrhosis; HC, healthy controls; PAI-1, plasminogen activator inhibitor-1; SC, stable cirrhosis; tPA, tissue-type plasminogen activator.

### 3.3 Lipid parameters, except for triglycerides, progressively decrease with cirrhosis severity

To quantify lipid profiles, we measured serum levels of common lipoproteins and their respective apolipoproteins (Figure 2), and levels of total cholesterol and triglycerides (Figure 3). Levels of apolipoprotein A1 and apolipoprotein B decreased progressively with cirrhosis severity compared with controls, and were similar in patients with decompensated cirrhosis and sepsis. Compared with controls, HDL, LDL, VLDL, and total cholesterol levels were lower in patients with stable cirrhosis, decompensated cirrhosis, ACLF, and sepsis, except for HDL and total cholesterol in patients with decompensated cirrhosis and VLDL in patients with stable cirrhosis. Lipoprotein(a) levels were relatively preserved in patients with stable cirrhosis, decompensated cirrhosis, and sepsis but lower in patients with ACLF than in controls. Similarly, triglyceride levels were preserved in all cirrhotic subgroups but were higher in patients with sepsis than in controls. As previous studies found elevated triglyceride levels in patients with underlying alcohol-related liver disease and MASLD, and 88% of our patients had either etiology, we hypothesized preserved levels to be related to the type of etiology. However, triglyceride levels did not significantly differ among different cirrhosis etiologies (Supplementary Figure).

**Figure 2:**
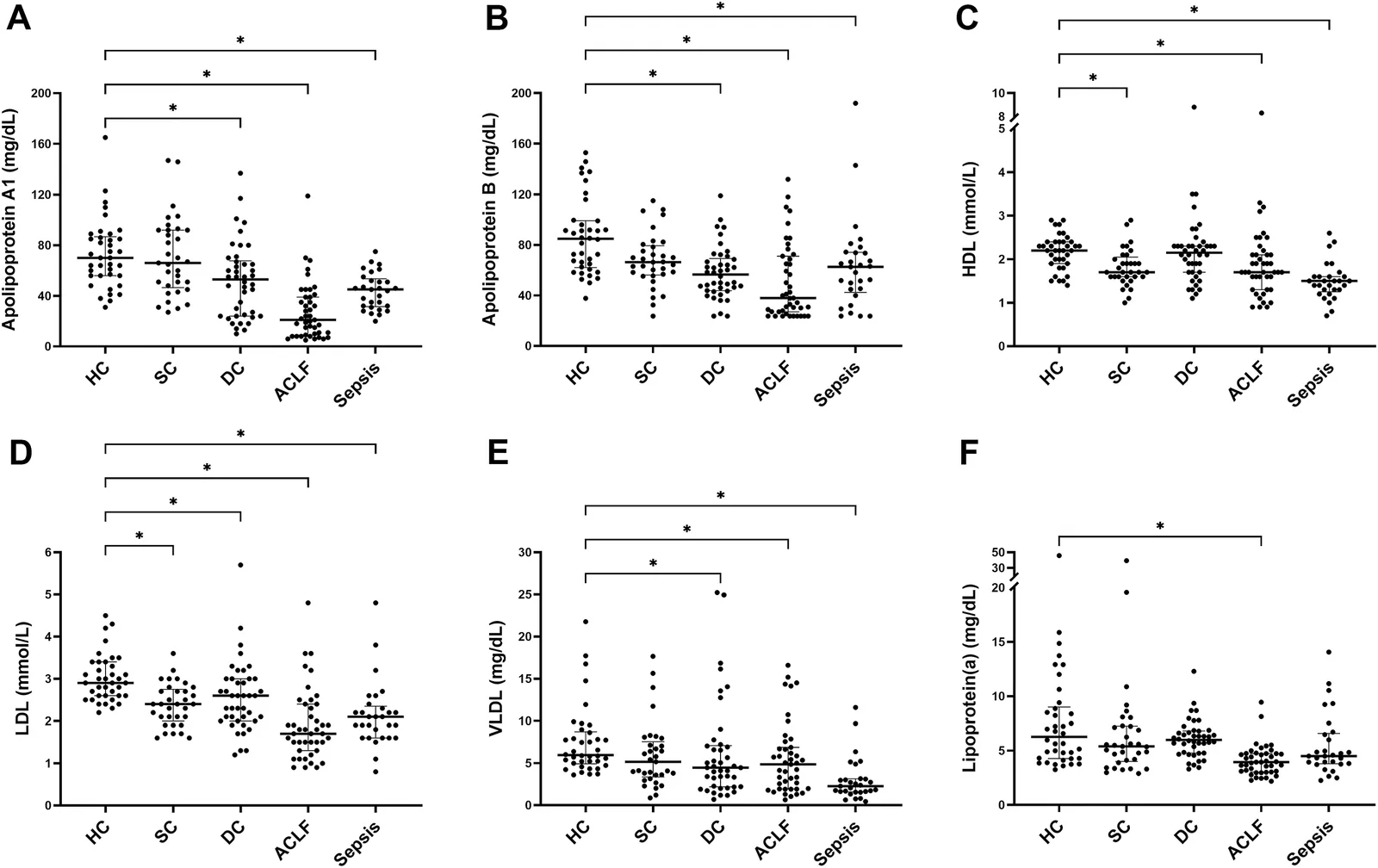
Serum (apo)lipoprotein levels. Apolipoprotein A1 (A), apolipoprotein B (B), HDL (C), LDL (D), VLDL (E), and lipoprotein(a) (F) were measured in patients with stable cirrhosis (*n* = 33), decompensated cirrhosis (*n* = 42), ACLF (*n* = 43), or sepsis without pre-existing liver disease (*n* = 29), compared with healthy controls (*n* = 38). The lines with error bars indicate medians with IQRs. ∗*P* < .05 all vs healthy controls using the Kruskal-Wallis test with Dunn’s multiple comparisons test or Brown-Forsythe and Welch’s 1-way analysis of variance with Dunnett’s T3 multiple comparisons. ACLF, acute-on-chronic liver failure; DC, decompensated cirrhosis; HC, healthy controls; HDL, high-density lipoprotein cholesterol; LDL, low-density lipoprotein cholesterol; SC, stable cirrhosis; VLDL, very-low-density lipoprotein cholesterol.

**Figure 3:**
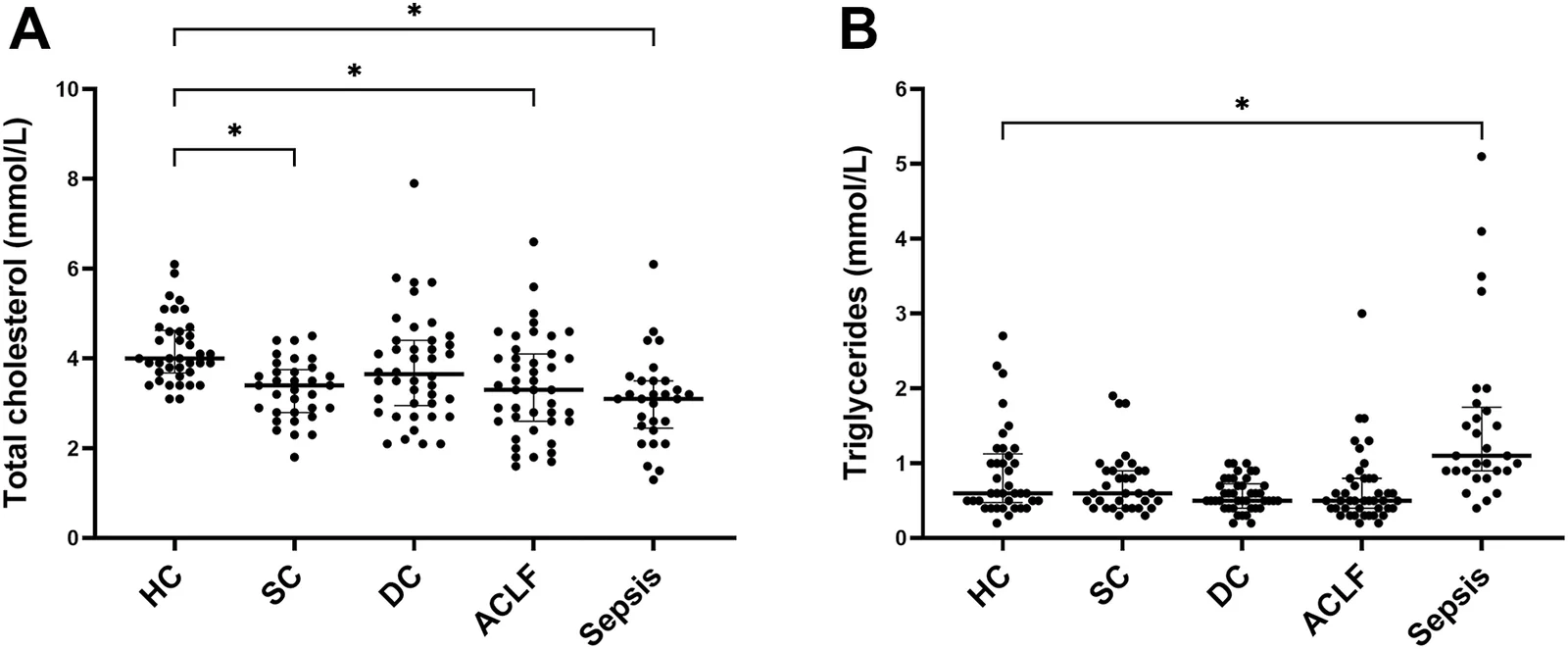
Serum lipid levels. Serum lipid parameters of total cholesterol (A) and triglycerides (B) were measured in patients with stable cirrhosis (*n* = 33), decompensated cirrhosis (*n* = 42), ACLF (*n* = 43), or sepsis without pre-existing liver disease (*n* = 29), compared with healthy controls (*n* = 38). The lines with error bars indicate medians with IQRs. ∗*P* < .05 all vs healthy controls using the Kruskal-Wallis test with Dunn’s multiple comparisons test or Brown-Forsythe and Welch’s 1-way analysis of variance with Dunnett’s T3 multiple comparisons. ACLF, acute-on-chronic liver failure; DC, decompensated cirrhosis; HC, healthy controls; SC, stable cirrhosis.

### 3.4 Abnormal lipid profiles are associated with markers of impaired fibrinolysis in patients with cirrhosis

To assess potential associations between lipid profiles and fibrinolytic parameters in patients with cirrhosis (*n* = 118) and patients with sepsis (*n* = 29), we performed predefined correlation analyses with adjustment for multiple comparisons. In patients with cirrhosis, apolipoprotein A1 levels were inversely associated with CLT, PAI-1, and tPA, and positively with plasminogen levels (Figure 4). Levels of apolipoprotein B, LDL, and lipoprotein(a) were positively associated with plasminogen levels, and triglyceride levels were positively associated with CLT (Figure 5). All other correlations remained statistically nonsignificant after adjustment for multiple comparisons in patients with cirrhosis (Supplementary Table 1) and in patients with sepsis (Supplementary Table 2).

**Figure 4:**
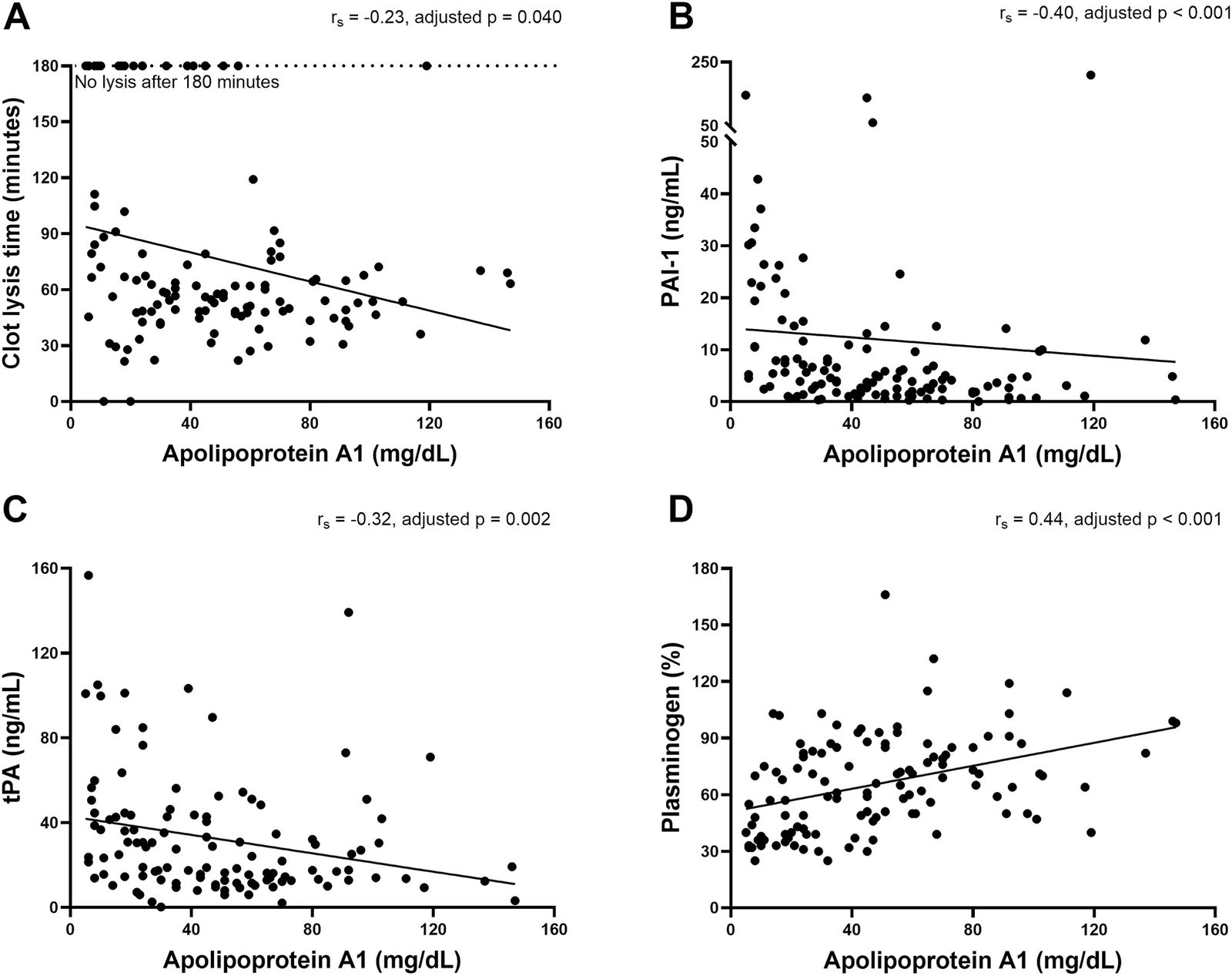
Associations of apolipoprotein A1 with fibrinolytic markers. Correlations of apolipoprotein A1 with clot lysis time (A), PAI-1 (B), tPA (C), and plasminogen (D) in patients with cirrhosis (*n* = 118). Shown are linear regression lines and Spearman’s rank correlation coefficients (*r_s_*) with the corresponding adjusted *P* values (false discovery rate adjusted using the 2-stage linear step-up procedure of Benjamini, Krieger, and Yekutieli at *Q*: 5%). Only statistically significant correlations after false discovery rate adjustment are depicted. PAI-1, plasminogen activator inhibitor-1; tPA, tissue-type plasminogen activator.

**Figure 5:**
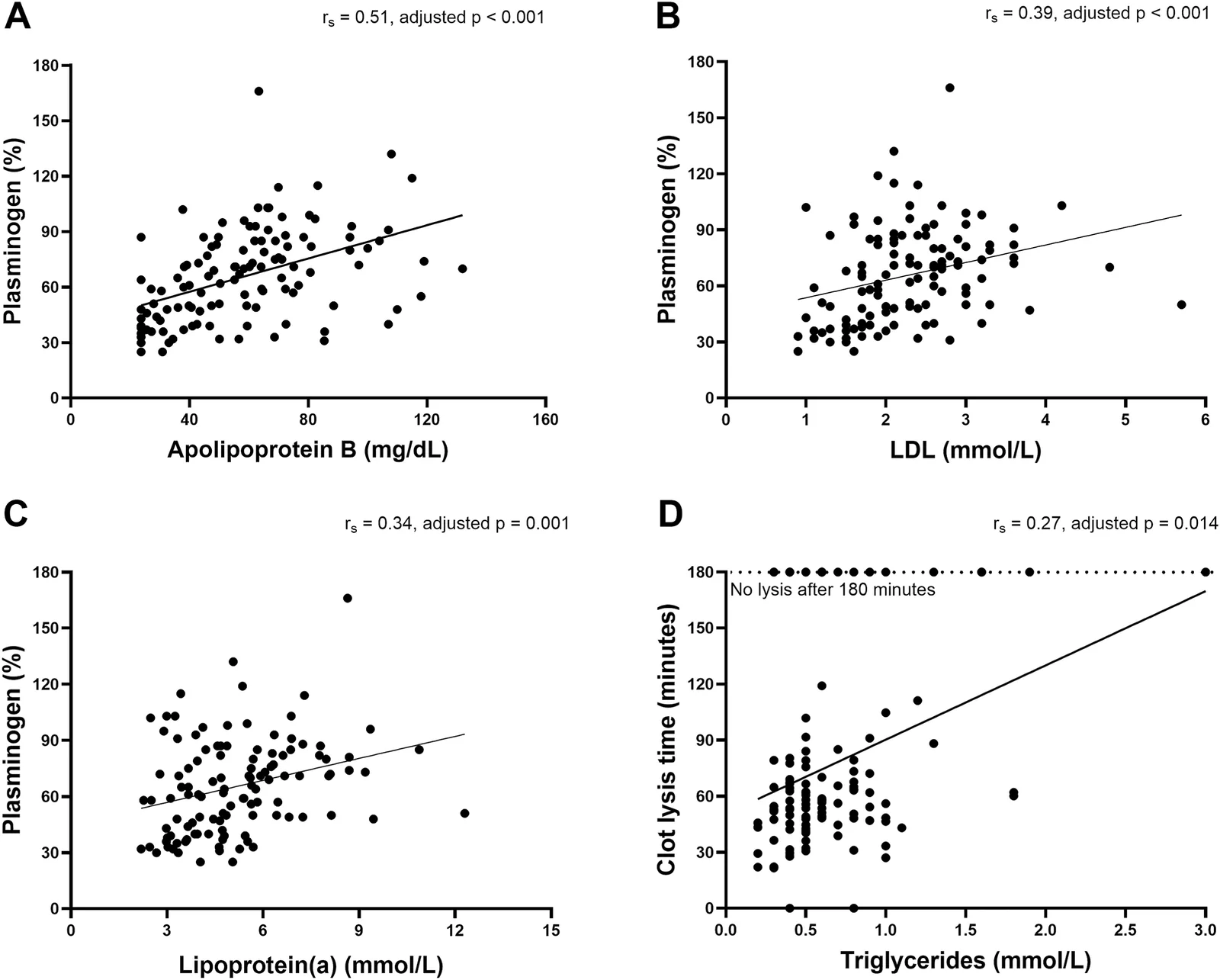
Associations of apolipoprotein B, LDL, lipoprotein(a), and triglycerides with fibrinolytic markers. Correlations of apolipoprotein B (A), LDL (B), and lipoprotein(a) (C) with plasminogen, and of clot lysis time with triglycerides (D), in patients with cirrhosis (*n* = 118). Shown are linear regression lines and Spearman’s rank correlation coefficients (*r_s_*) with the corresponding adjusted *P* values (false discovery rate adjusted using the 2-stage linear step-up procedure of Benjamini, Krieger, and Yekutieli at *Q*: 5%). Only statistically significant correlations after false discovery rate adjustment are depicted. LDL, low-density lipoprotein.

## 4 Discussion

In this observational study, we found decreased (apo)lipoprotein and preserved triglyceride levels, and a shift from enhanced to impaired fibrinolytic capacity across progressive stages of cirrhosis. We identified several associations between lipid profiles and fibrinolytic capacity. In particular, lower apolipoprotein A1 and higher triglyceride levels correlated with prolonged CLT, which aligns with previous experimental data that suggest direct interactions between apolipoprotein A1 and triglyceride levels, and fibrinolysis [18,20,21,27,28]. Together, our data confirm the transition from enhanced to impaired fibrinolysis [3,4], extend current knowledge on lipid abnormalities [[29], [30], [31], [32]], and identify potential links between lipid abnormalities and reduced fibrinolytic capacity in patients with cirrhosis.

In line with prior studies, fibrinolytic capacity was normal to slightly enhanced in patients with stable cirrhosis and decompensated cirrhosis, as reflected by a shortened CLT [4], which is likely driven by elevated tPA activity relative to PAI-1 activity [3]. In contrast, fibrinolysis was impaired in patients with ACLF and sepsis, which is also consistent with earlier reports [4]. Both portal hypertension and systemic inflammation likely promote endothelial activation, resulting in increased PAI-1 release and elevated circulating levels in patients with ACLF and sepsis [26].

We observed a progressive decrease in (apo)lipoprotein levels in patients with cirrhosis, as demonstrated by low levels of apolipoprotein A1, apolipoprotein B, HDL, LDL, VLDL, lipoprotein(a), and total cholesterol across cirrhosis severity. Our data on (apo)lipoprotein level reductions concur with and extend decade-old studies [[29], [30], [31],^33^], providing a comprehensive and clinically stratified overview of lipid profiles in patients with cirrhosis. The progressive decrease in (apo)lipoprotein levels is consistent with impaired hepatic lipoprotein synthesis, assembly, and secretion. Consistent with previous studies [34,35], apolipoprotein A1 and HDL were particularly low in patients with ACLF and sepsis. This likely occurred as a consequence of systemic inflammation-related remodeling and consumption of HDL, as apolipoprotein A1 within HDL particles binds and neutralizes bacterial endotoxins [36].

In contrast to most previous studies [23], we found relatively preserved triglyceride levels in patients with cirrhosis, with a trend toward lower levels in those with decompensated cirrhosis and ACLF than in stable cirrhosis and controls. One explanation could be that alcohol-related liver disease and MASLD have been shown to increase hepatic triglyceride synthesis and peripheral lipolysis, modulate lipogenic enzymes, and inhibit fatty acid oxidation [24,25]. However, although these etiologies were present in the vast majority of our patients, we did not find significant differences in triglyceride levels compared with other etiologies. An alternative explanation might be acquired hepatic triglyceride lipase deficiency secondary to impaired hepatic function, resulting in increased triglyceride levels. Prior studies have shown hepatic triglyceride lipase deficiency to be present in cirrhosis and earlier stages of liver disease [37]. In addition, insulin resistance is common in patients with cirrhosis and might contribute to increases in triglyceride levels despite hepatic synthetic capacity [38]. Finally, preserved triglyceride levels may have been related to nutritional status, as fasting status was not recorded, and recent dietary intake might have elevated initially decreased triglyceride levels.

Multiple lipid parameters were associated with markers of fibrinolysis in patients with cirrhosis, as indicated by inverse correlations of apolipoprotein A1 and positive correlations of apolipoprotein B, LDL, lipoprotein(a), and triglycerides with impaired clot breakdown. These correlations may reflect biological interactions, parallel consequences of cirrhosis severity, a combination of both, or other unaccounted mechanisms. Importantly, we did not perform analyses adjusted for cirrhosis severity because it may be integral to the biological pathways linking lipid and fibrinolytic parameters. Such adjustment could therefore result in statistical overadjustment and obscure rather than clarify the observed correlations. Our observations are partly in line with previous mechanistic studies in humans and mice without liver disease, as discussed below. Importantly, correlations observed in this cross-sectional study cannot establish mechanistic relationships and should not be considered independent of cirrhosis severity, and future studies will need to further examine potential causal interactions.

Mice with apolipoprotein A1 deficiency show higher rates of stasis-induced venous thrombosis than wild-type mice, and apolipoprotein A1 infusion rescues wild-type mice, but not mice with deficiency in scavenger receptor B type 1 or endothelial nitric oxide synthase, from this prothrombotic phenotype [21]. Hence, lack of apolipoprotein A1–induced, scavenger receptor B1–mediated activation of endothelial nitric oxide synthase results in less production of nitric oxide [21,39]. Lack of nitric oxide induces endothelial dysfunction and activation [40], and may thereby result in elevated PAI-1 levels, which are known to increase risk of venous thrombosis by inhibiting clot breakdown [41]. Further supporting these data, apolipoprotein A1 activates fibrinolysis in purified systems, although underlying mechanisms have not been studied in detail [27]. Moreover, apolipoprotein A1 has been shown to exert *in vitro* anticoagulant effects through HDL-enhanced activation of protein C and S, which may indirectly improve fibrinolysis through effects on fibrin structure and activation of thrombin activatable fibrinolysis inhibitor [[42], [43], [44]].

Similar to apolipoprotein A1, triglycerides may impair fibrinolysis primarily through increased PAI-1 expression. Free fatty acids are the breakdown products of triglycerides and have been shown to increase PAI-1 expression in adipose tissue macrophages and endothelial cells [28,45]. In addition, free fatty acids may promote expression of tumor necrosis factor-alpha through cathepsin B–dependent lysosomal destabilization [46]. In turn, this has been demonstrated to increase PAI-1 expression in adipose tissue in mice [47]. VLDL, the primary transporter of triglycerides, also induces PAI-1 synthesis and transcription in HepG2 cells and endothelial cells [48,49], although VLDL itself did not show statistically significant correlations with PAI-1 in our study. Despite these plausible mechanisms, the clinical relevance of the association between triglycerides and prolonged CLT in cirrhosis may be limited due to the absence of marked hypertriglyceridemia in our patients, restricting the potential relevance of triglyceride-lowering drugs to improve fibrinolysis and reduce thrombotic risk.

Lipoprotein(a) is another parameter that might influence fibrinolysis. The apolipoprotein(a) subunit of lipoprotein(a) structurally resembles plasminogen with similar kringle domains that competitively bind fibrin [22]. Thereby, lipoprotein(a) may reduce plasminogen binding, impair fibrinolysis, and potentially increase thrombotic risk [19]. Although both lipoprotein(a) and apolipoprotein(a) have been shown to prolong CLT *ex vivo* in recent studies [19], these data have been debated in other literature [50]. In addition, lowering the production of apolipoprotein(a) using an antisense oligonucleotide (IONIS-APO(a)Rx/Pelacarsen, Ionis Pharmaceuticals) in humans showed no differences in CLT in patients compared with placebo [50]. Although these studies suggest interactions between lipoprotein(a) and plasminogen, they may be less important for patients with cirrhosis [31]. As our study indicates that both lipoprotein(a) and plasminogen substantially decrease in patients with cirrhosis, the overall effect of lipoprotein(a) on impairing fibrinolysis through interactions with plasminogen may be less important.

Besides potential interactions between lipids and fibrinolytic parameters, the observed correlations may partly reflect shared effects of impaired hepatic synthetic function and increased endothelial activation. Hepatic protein synthesis progressively declines with increasing cirrhosis severity, resulting in reduced levels of liver-derived lipids, (apo)lipoproteins, and plasminogen [23,30]. Associations such as those of apolipoprotein B and LDL with plasminogen may therefore reflect a common impairment in hepatic dysfunction rather than direct interactions. PAI-1 and tPA are released primarily by endothelial cells, and their levels may increase because of progressive endothelial activation associated with portal hypertension and systemic inflammation in cirrhosis [2,3,51]. However, prior studies report correlations of hepatocyte–but not endothelial-derived hemostatic proteins with apolipoproteins [13]. Collectively, our findings may suggest potential interactions between lipid abnormalities and impaired fibrinolysis, particularly for low apolipoprotein A1 and high triglyceride levels. Nevertheless, some associations may reflect parallel cirrhosis-related changes in hepatic protein and lipid synthesis and increased endothelial activation.

Strengths of this study included a well-defined, clinically stratified group of cirrhotic patients from a prospective observational cohort study with recruitment from a high-volume liver center. We performed extensive profiling of lipids and (apo)lipoproteins, providing a comprehensive overview of lipid parameters in cirrhosis stages based on most up-to-date clinical stratifications. To minimize false-positive correlations as a consequence of performing multiple testing, we adjusted for multiple comparisons using the Benjamini, Krieger, and Yekutieli method to control the false discovery rate.

This study has several limitations that should be considered when interpreting the findings. First, the observational design precludes causal inference and does not allow assessment of directionality. Second, although the study included a well-defined patient cohort from a high-volume liver center, the findings were not externally validated. Future studies should aim to validate our results in independent cohorts, ideally through multicenter collaborations or national registry-based analyses, to assess generalizability. Third, information on statin use was unavailable for almost half of the patients and was not collected for healthy controls. This aspect may have introduced residual confounding, as statins not only lower lipid concentrations but also influence fibrinolysis and liver disease progression. The extent and direction of any resulting bias are difficult to predict; however, statin use was infrequent among patients with available information and was likely uncommon among relatively young healthy controls. Fourth, fasting status at the time of blood sampling was not recorded, and an effect of recent dietary intake on baseline lipid measurements cannot be fully excluded. This effect was likely limited, because lipid profiles were consistently low across patients with cirrhosis and were comparable to those reported in prior studies that included fasted participants [30]. Finally, age- and sex-related differences between healthy controls and patients may have introduced residual confounding, although the pattern of the observed differences supports, yet does not establish, a predominant contribution of liver disease severity in line with previous literature [4,52,53].

## 5 Conclusion

In conclusion, lipid profiles are associated with fibrinolytic parameters in patients with cirrhosis, supported by profound decreases in (apo)lipoprotein levels, preserved triglyceride levels, and progression from enhanced to impaired fibrinolysis. In particular, the associations between low apolipoprotein A1 and high triglyceride levels with prolonged CLT may indicate biological interactions, in line with previous observational and experimental studies. However, these correlations may also reflect simultaneous changes in response to progressing hepatic dysfunction. Our results should be regarded as hypothesis-generating, and future studies should investigate whether these associations are confirmed by mechanistic experiments in cirrhosis using cell- or animal-based models, for example, by assessing the effect of apolipoprotein A1 knockout on *ex vivo* fibrinolysis or venous thrombosis in rodent models of cirrhosis.
