# Supplementary material for: Abnormal lipid profiles are associated with markers of impaired fibrinolysis in patients with cirrhosis
Source: Res Pract Thromb Haemost. 2026 Sep 18;10(7):106975. doi: 10.1016/j.rpth.2026.106975 (PMC13677304; doi:10.1016/j.rpth.2026.106975)
Supplement: Supplementary file 1 [file mmc1.docx]

**Supplementary material to: Abnormal lipid profiles are associated with markers of impaired fibrinolysis in patients with cirrhosis**

**Authors**

Fynn L. Elvers^1^, Jelle Adelmeijer^1^, Marilena Stamouli^2^, William Bernal^3^, Vishal C. Patel^2^, Ton Lisman^1^

**Corresponding author**

Ton Lisman, Surgical Research Laboratory, Department of Surgery, University Medical Center Groningen, BA33, Hanzeplein 1, 9713 GZ Groningen, The Netherlands. Tel: +31 6 25 64 86 64. Fax: +31-50-363-2796.

**Content**

[*Figures	2*](#_Toc236835362)

[*Figure S1. Triglyceride levels across etiologies of cirrhosis.	2*](#_Toc236835363)

[*Tables	3*](#_Toc236835364)

[*Table S1. Non-significant correlations of lipid profiles with fibrinolytic markers in patients with cirrhosis.	3*](#_Toc236835365)

[*Table S2. Non-significant correlations of lipid profiles with fibrinolytic markers in patients with sepsis without underlying liver disease	5*](#_Toc236835366)

# **Figures**

**Figure S1. Triglyceride levels across etiologies of cirrhosis.** Shown are median levels as bars with individual patient data points. No statistically significant differences in triglyceride levels were found between different etiologies using Kruskal-Wallis test with Dunn’s multiple comparisons test. Others: Hemochromatosis, Budd-Chiari, cryptogenic cirrhosis, biliary atresia. Abbreviations: ALD, alcoholic liver disease; MASH/MASLD, metabolic dysfunction-associated steatohepatitis/metabolic dysfunction-associated steatotic liver disease; HCV, hepatitis C virus; PSC/PBC/AIH, primary sclerosing cholangitis/primary biliary cholangitis/autoimmune hepatitis.

# **Tables**

## **Table S1. Non-significant correlations of lipid profiles with fibrinolytic markers in patients with cirrhosis.**

| **Lipid profiles** | **Fibrinolytic markers** | **Spearman’s r_s_** | **Raw p-value** | **Adjusted p-value** |
| --- | --- | --- | --- | --- |
| Apolipoprotein B | Clot lysis time | 0.10 | 0.290 | 0.475 |
| Apolipoprotein B | PAI-1 | 0.00 | 0.985 | 0.808 |
| Apolipoprotein B | tPA | -0.08 | 0.378 | 0.537 |
| HDL | Clot lysis time | 0.02 | 0.813 | 0.762 |
| HDL | PAI-1 | 0.00 | 0.983 | 0.808 |
| HDL | tPA | 0.07 | 0.441 | 0.539 |
| HDL | Plasminogen | 0.06 | 0.502 | 0.553 |
| LDL | Clot lysis time | -0.07 | 0.452 | 0.539 |
| LDL | PAI-1 | -0.16 | 0.079 | 0.183 |
| LDL | tPA | -0.17 | 0.058 | 0.154 |
| VLDL | Clot lysis time | -0.09 | 0.308 | 0.475 |
| VLDL | PAI-1 | 0.08 | 0.389 | 0.537 |
| VLDL | tPA | -0.06 | 0.506 | 0.553 |
| VLDL | Plasminogen | 0.08 | 0.411 | 0.539 |
| Lipoprotein(a) | Clot lysis time | -0.01 | 0.930 | 0.808 |
| Lipoprotein(a) | PAI-1 | -0.05 | 0.609 | 0.592 |
| Lipoprotein(a) | tPA | -0.13 | 0.171 | 0.320 |
| Total cholesterol | Clot lysis time | 0.10 | 0.280 | 0.475 |
| Total cholesterol | PAI-1 | 0.06 | 0.529 | 0.555 |
| Total cholesterol | tPA | 0.05 | 0.575 | 0.581 |
| Total cholesterol | Plasminogen | 0.16 | 0.084 | 0.183 |
| Triglycerides | PAI-1 | 0.15 | 0.111 | 0.225 |
| Triglycerides | tPA | 0.01 | 0.915 | 0.808 |
| Triglycerides | Plasminogen | 0.22 | 0.019 | 0.055 |

Correlations were calculated for each pair of lipids and fibrinolytic markers for pooled cirrhotic patients (n = 118) including stable cirrhosis, decompensated cirrhosis and acute‐on-chronic liver failure. Shown are Spearman’s rank correlation coefficients (r) with the corresponding raw p-values and adjusted p-values (FDR adjusted using the two-stage linear step-up procedure of Benjamini, Krieger and Yekutieli at Q: 5%). Only statistically non-significant correlations after FDR adjustment are shown.

Abbreviations: HDL, high-density lipoprotein; FDR, false discovery rate; LDL, low-density lipoprotein; PAI-1, plasminogen activator inhibitor-1; tPA, tissue-type plasminogen activator; VLDL, very-low-density lipoprotein.

## **Table S2. Non-significant correlations of lipid profiles with fibrinolytic markers in patients with sepsis without preexisting chronic liver disease**

| **Lipid profiles** | **Fibrinolytic markers** | **Spearman’s r_s_** | **Raw p-value** | **Adjusted p-value** |
| --- | --- | --- | --- | --- |
| Apolipoprotein A1 | Clot lysis time | -0.18 | 0.363 | 0.581 |
| Apolipoprotein A1 | PAI-1 | 0.27 | 0.153 | 0.395 |
| Apolipoprotein A1 | tPA | -0.24 | 0.216 | 0.484 |
| Apolipoprotein A1 | Plasminogen | 0.14 | 0.457 | 0.698 |
| Apolipoprotein B | Clot lysis time | -0.08 | 0.690 | 0.799 |
| Apolipoprotein B | PAI-1 | -0.47 | 0.010 | 0.100 |
| Apolipoprotein B | tPA | -0.31 | 0.098 | 0.300 |
| Apolipoprotein B | Plasminogen | 0.45 | 0.014 | 0.100 |
| HDL | Clot lysis time | 0.23 | 0.240 | 0.503 |
| HDL | PAI-1 | -0.09 | 0.656 | 0.787 |
| HDL | tPA | 0.18 | 0.354 | 0.581 |
| HDL | Plasminogen | 0.33 | 0.084 | 0.300 |
| LDL | Clot lysis time | 0.02 | 0.900 | 0.972 |
| LDL | PAI-1 | -0.31 | 0.098 | 0.300 |
| LDL | tPA | -0.12 | 0.539 | 0.780 |
| LDL | Plasminogen | 0.48 | 0.009 | 0.100 |
| VLDL | Clot lysis time | -0.02 | 0.926 | 0.972 |
| VLDL | PAI-1 | -0.26 | 0.168 | 0.403 |
| VLDL | tPA | -0.31 | 0.098 | 0.300 |
| VLDL | Plasminogen | 0.37 | 0.048 | 0.230 |
| Lipoprotein(a) | Clot lysis time | -0.09 | 0.627 | 0.780 |
| Lipoprotein(a) | PAI-1 | -0.21 | 0.274 | 0.541 |
| Lipoprotein(a) | tPA | -0.18 | 0.351 | 0.581 |
| Lipoprotein(a) | Plasminogen | 0.45 | 0.015 | 0.100 |
| Total cholesterol | Clot lysis time | 0.04 | 0.852 | 0.954 |
| Total cholesterol | PAI-1 | -0.19 | 0.320 | 0.581 |
| Total cholesterol | tPA | -0.11 | 0.571 | 0.780 |
| Total cholesterol | Plasminogen | 0.52 | 0.004 | 0.100 |
| Triglycerides | Clot lysis time | 0.10 | 0.610 | 0.780 |
| Triglycerides | PAI-1 | -0.30 | 0.118 | 0.331 |
| Triglycerides | tPA | -0.11 | 0.588 | 0.780 |
| Triglycerides | Plasminogen | 0.40 | 0.030 | 0.169 |

Correlations were calculated for each pair of lipids and fibrinolytic markers for patients with sepsis without underlying liver disease (n = 29). Shown are Spearman’s rank correlation coefficients (r) with the corresponding raw p-values and adjusted p-values (False discovery rate adjusted using the two-stage linear step-up procedure of Benjamini, Krieger and Yekutieli at Q: 5%). Only statistically non-significant correlations after FDR adjustment are shown.

Abbreviations: HDL, high-density lipoprotein; LDL, low-density lipoprotein; PAI-1, plasminogen activator inhibitor-1; tPA, tissue-type plasminogen activator; VLDL, very-low-density lipoprotein.
